# Long‐term Tai Chi practice in older adults is associated with “younger” functional abilities

**DOI:** 10.1111/acel.14023

**Published:** 2023-10-31

**Authors:** Yan Ma, Brian J. Gow, Rhayun Song, Pamela M. Rist, Jeffrey M. Hausdorff, Lewis A. Lipsitz, Brad Manor, Peter M. Wayne

**Affiliations:** ^1^ Osher Center for Integrative Health, Division of Preventive Medicine Brigham and Women's Hospital and Harvard Medical School Boston Massachusetts USA; ^2^ Laboratory for Computational Physiology (LCP) Massachusetts Institute of Technology Cambridge Massachusetts USA; ^3^ College of Nursing Chungnam National University Daejeon Korea; ^4^ Division of Preventive Medicine Brigham and Women's Hospital, Harvard Medical School Boston Massachusetts USA; ^5^ Center for the Study of Movement, Cognition, and Mobility, Neurological Institute, Tel Aviv Sourasky Medical Center; Sagol School of Neuroscience and Department of Physical Therapy, Sackler Faculty of Medicine Tel Aviv University Tel Aviv Israel; ^6^ Department of Orthopedic Surgery and Rush Alzheimer's Disease Center Rush University Medical Center Chicago Illinois USA; ^7^ Hinda and Arthur Marcus Institute for Aging Research Hebrew SeniorLife Boston Massachusetts USA; ^8^ Division of Gerontology, Beth Israel Deaconess Medical Center Harvard Medical School Boston Massachusetts USA

**Keywords:** aging, balance and fall, physical function, Tai Chi

## Abstract

Age‐related alterations in physiology lead to declines in physical function that are associated with numerous adverse outcomes among older adults. Utilizing a hybrid design, we aimed to understand whether both long‐term and short‐term Tai Chi (TC) training are associated with age‐related decline in physical function in healthy older adults. We first conducted cross‐sectional comparisons among TC‐naïve older adults (*n* = 60, 64.2 ± 7.7 years), TC‐expert older adults (*n* = 27, 62.8 ± 7.6 years, 24.5 ± 12 years experience), and TC‐naïve younger adults (*n* = 15, 28.7 ± 3.2 years) to inform long‐term effects of TC training on physical function, including single leg stance time with eyes closed, grip strength, Timed Up and Go, maximum walking speed, functional reach, and vertical jump for lower‐extremity power. There were significant differences among the three groups on all the six tests. For most functional tests, TC‐experts performed better than age‐matched TC‐naïve controls and were statistically indistinguishable from young healthy adult controls. Long‐term TC training was associated with higher levels of physical function in older adults, suggesting a potential preventative healthy aging effect. In the randomized longitudinal trial, TC‐naïve subjects were randomized (*n* = 31 to Tai Chi group, *n* = 29 to usual care control group) to evaluate the short‐term effects of TC over 6 months on all outcomes. TC's short‐term impacts on physical function were small and not statistically significant. The impact of short‐term training in healthy adults is less clear. Both potential longer‐term preventive effects and shorter‐term restorative effects warrant further research with rigorous, adequately powered controlled clinical trials.

## Graphical abstract

There were significant differences among the three groups on all the six tests. For most functional tests, Tai Chi‐experts performed better than age‐matched Tai Chi‐naïve controls and were statistically indistinguishable from young healthy adult controls. Long‐term Tai Chi training was associated with higher levels of physical function in older adults, suggesting a potential preventative healthy aging effect.

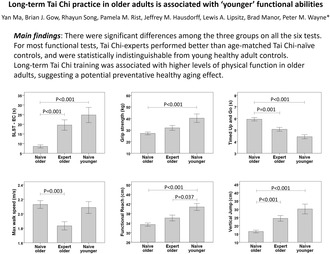

**Abbreviations**
MWS: maximum walking speed
RCT: randomized clinical trial
SLST‐EC: single leg stance time during eyes‐closed
TC: Tai Chi
TUG: Timed Up and Go

## 1 INTRODUCTION

Aging is associated with declines in multiple domains of physical function including mobility, balance, and strength. Age‐related functional decline, in turn, is associated with numerous adverse outcomes among older adults, including increased risks of falls and cardiovascular events, social isolation, increased hospitalizations and admissions to nursing homes, and overall reduced quality of life. Consequently, age‐related functional decline has a significant impact on societal health care costs, and these burdens are expected to grow with our aging society. Research informing effective interventions that prevent functional decline and promote healthy aging is therefore warranted.

A growing body of research supports the idea that exercise can be effective in preventing functional decline. However, optimal types and regimens of exercise that are effective, safe, adaptable, enjoyable, and cost‐effective and thus likely to be adopted and adhered to by older adults across the later lifespan have yet to be systematically evaluated. Tai Chi is a mind–body exercise that has gained much attention in recent years as a putative preventive and rehabilitative exercise option for a wide range of clinical populations. With roots in traditional Chinese martial and healing arts, Tai Chi employs detailed regimens of flowing movements, upright balance and weight shifting, breathing techniques, and cognitive tools (e.g., focused attention, imagery, and body awareness). Studies have investigated Tai Chi's effects on multiple outcomes that are associated with morbidity including cardiopulmonary conditions, musculoskeletal pain (Hall et al., 2017), poor balance, compromised immune function, cognition, and mental health. In addition to promising clinical outcomes, research suggests that Tai Chi is easily adaptable/modifiable for different abilities, including for sedentary, deconditioned individuals starting an exercise program. Tai Chi also enhances exercise self‐efficacy and can serve as a bridge to more broad engagement in physical activity. This adaptability and appeal may underlie reports of higher adherence in clinical trials, compared to more conventional exercises, and the potential for lifelong learning and long‐term sustainability of Tai Chi.

While Tai Chi's impact on clinical populations has received significant attention, less research has focused on the potential benefits for healthy and relatively active adult populations. Some of this evidence gap results from the general challenges of studying upstream preventative interventions, including pragmatic issues related to the delivery of longer‐term nonpharmacological interventions and extended observational periods (Ninot, 2021). As one strategy for simultaneously exploring both longer‐ and shorter‐term effects of Tai Chi in healthy adults in a coordinated fashion, we have developed and employed a novel hybrid‐design approach (Wayne et al., 2013). Using this approach, potential longer‐term training effects are assessed through observational comparisons between Tai Chi‐naive healthy older adults and an age‐matched sample of highly trained Tai Chi experts. Employing identical outcome assessment protocols, short‐term effects are assessed by random assignment of the Tai Chi‐naive healthy adults to either 6 months of Tai Chi training plus usual care versus usual care alone. Prior publications using this design have reported on the long‐ and short‐term effects of Tai Chi on postural control assessed through center of pressure dynamics (Wayne et al., 2013), gait dynamics (Gow et al., 2017; Wayne et al., 2015), and cognitive function (Jor'dan et al., 2018; Walsh et al., 2015). In this study, we extend our use of a hybrid design and evaluation of the same study population in two ways. First, we report on the impact of Tai Chi on a battery of functional measures commonly evaluated in older adult populations. Second, as a reference point for characterizing age‐related decline in functional measures and for understanding the potential long‐term effects of Tai Chi on such decline, we include a second younger (age 25–35 years) Tai Chi‐naïve group.

Based on our prior studies and existing literature, we hypothesized that: (1) Tai Chi experts would show better physical function outcomes relative to age‐matched Tai Chi‐naive older adults; (2) Relative to Tai Chi‐naive older adults, Tai Chi experts would have functional measures more similar to Tai Chi‐naïve healthy, young adults (i.e., greater attenuation of functional decline); (3) Within the embedded randomized trial, older adults randomized to the short‐term Tai Chi training group would exhibit better functional outcomes than those in the usual care control group after 6 months of Tai Chi training.

## 2 METHODS

We employed a hybrid study design that included a two‐arm randomized clinical trial (RCT) along with two additional observational comparison groups. The Institutional Review Board at Beth Israel Deaconess Medical Center (BIDMC) approved this study. The RCT component of this study was registered at clinicaltrials.gov (NCT01340365). Physical function outcomes reported herein are a subset of a larger battery of assessed outcomes including gait, balance, cardiovascular, and cognitive outcomes. These latter outcomes are reported independently elsewhere (Gow et al., 2017; Jor'dan et al., 2018; Ma et al., 2019; Walsh et al., 2015; Wayne et al., 2013, 2014, 2015, 2021).

### 2.1 Randomized trial design

A total of 60 Tai Chi‐naïve healthy older adults, aged 50–79, were randomized to receive 6 months of Tai Chi training in addition to usual care or to usual health care alone (control group). Participants randomized to usual care were offered a 3‐month course of Tai Chi as a courtesy following the trial. Randomization was stratified by age (50–59 years, 60–69 years, and 70–79 years) and utilized a permuted‐blocks randomization scheme with randomly‐varying block sizes. All outcomes were assessed at baseline, 3 months, and 6 months; primary staff overseeing assessment and analysis of gait‐related outcomes were blinded to treatment assignment. Further details related to the design of the RCT component of this study are reported elsewhere (Wayne et al., 2013).

### 2.2 Recruitment for the randomized trial targeting community‐dwelling healthy adults

Inclusion criteria were as follows: (1) Age 50–79 years; (2) living or working within the Greater Boston area; and (3) willing to adhere to a 6‐month Tai Chi training protocol. Exclusion criteria were as follows: (1) Chronic medical condition including cardiovascular disease (myocardial infarction, angina, atrial fibrillation, or presence of a pacemaker), stroke, respiratory disease requiring daily use of an inhaler; diabetes mellitus; active cancer (diagnosis <5 years ago and requiring ongoing chemotherapy or use of cytotoxic agents), stage III prostate cancer, dermatological cancer with reoccurrence; neurological conditions (e.g., seizure disorder, Parkinson's, and peripheral neuropathy); or significant neuromuscular or musculoskeletal conditions requiring chronic use of pain medication; (2) acute medical condition requiring hospitalization within the past 6 months; (3) self‐reported (current) smoking or alcohol/drug abuse; (4) uncontrolled hypertension (resting SBP > 160 or DBP > 100 mm Hg); (5) abnormal heart rate (resting HR > 100 bpm; < 50 bpm); (6) abnormal ECG (e.g., supraventricular tachyarrhythmia, atrial fibrillation, significant ST wave abnormality, 2nd and 3rd degree heart block); (7) pregnancy; (8) current use of cardio‐ or vaso‐active drugs and medications that can affect autonomic function including beta agonists and antagonists, drugs with anticholinergic properties (e.g., tricyclic antidepressants, or anti psychotics), and cholinesterase inhibitors; (9) self‐reported inability to walk continuously for 15 min unassisted; (10) regular Tai Chi practice within past 5 years; and (11) regular participation in physical exercise on average 4 or more times per week. Interested individuals underwent both an initial phone screen and an in‐person screen at the BIDMC Clinical Research Center. Eligible individuals provided written informed consent and underwent baseline testing prior to randomization.

Participants within both groups were encouraged to follow usual healthcare as prescribed by their primary physicians, with no limitations set by the study other than those implicit in the eligibility criteria listed above. Participants in the Tai Chi group received 6 months of Tai Chi training in addition to usual care. All Tai Chi interventions were administered pragmatically at one of five prescreened Tai Chi schools within the Greater Boston area that met specific guidelines described elsewhere (Wayne et al., 2013). Instructors were asked to teach using the same Tai Chi style, approach, and protocols employed for nonstudy, community participants. Participants were asked to attend, on average, two classes per week over the 6‐month intervention, and to practice a minimum of 30 min, two additional days per week. All schools provided DVDs or printed materials to facilitate home practice. In total, participants were asked to engage in 72 h of training over the 6‐month intervention. Attendance at Tai Chi classes was recorded by instructors, and home practice was tracked using a weekly practice log. Participants that reported attending a minimum of 70% of all classes and completing 70% or more of prescribed home practice between each study visit were considered compliant or “per protocol”.

### 2.3 Nonrandomized comparison groups

*Tai Chi experts*. Twenty‐seven healthy older adults (age 50–79 years) currently engaged in an active Tai Chi training regimen, and each with over 5 years of Tai Chi practice were recruited for a single observational visit. No limitation was set on Tai Chi style. The Tai Chi expert group was used to evaluate the longer‐term effects of Tai Chi training on physical function. Eligibility and screening procedures for Tai Chi experts were identical to those for healthy adults enrolled in the RCT, with the exceptions of no limitation on exercise activity or prior Tai Chi experience.

*Young Comparison Group*. Fifteen young Tai Chi‐naïve adults (age 25–35 years), meeting the healthy eligibility criteria outlined above, were recruited for a single observational visit. The group was used to better characterize age‐related trends in functional outcomes.

### 2.4 Measurements

*One‐legged standing balance with closed eyes* (Vereeck et al., 2008) has been correlated with fall risk in older adults (Omaña et al., 2021). Timing started when the subject had assumed the proper position on their preferred leg and verbally indicated readiness to begin the test. Timing stopped when the subject disengaged from the starting position (i.e., the suspended foot touches the floor or the hands touch support surface) or when the 30‐s time limit was reached. The test was conducted in a corner of a room, and the subject was instructed to reach the wall should they feel unstable. Three trials were completed for the single leg stance time (SLST) during eyes‐closed (EC) conditions, and the greatest duration (seconds) was used for analysis.

*Grip strength* is a widely used reliable measure correlated with mortality, disability, and overall function in middle‐aged and older adults (Bohannon, 2019). Grip strength was measured using a handgrip dynamometer Grip D (Takei Scientific Instruments, Tokyo, Japan). Subjects were asked to grip the instrument with their dominant hand with maximum force. Measurements were recorded to the nearest 0.5 kg, repeated three times and all recorded. The highest value was used in the analyses (Mehmet et al., 2020).

*Timed Up and Go (TUG) Test* is validated for quantifying functional mobility following clinical change over time (Christopher et al., 2021; Nightingale et al., 2019). Patients began in a seated position, stood up from a chair, walked a short distance (3 meters, ~10 feet) at a comfortable pace, turned around, returned, and sat down again. This test was timed by the blinded research assistant. Participants repeated the test and the times were recorded. The minimum TUG (in second) was used in this analysis. This quick test is easily included as part of the routine medical examination.

*Maximum Walking Speed* is a common, easily administered clinical test associated with functional decline (Rydwik et al., 2012; Shinkai et al., 2000). Subjects walked in a straight line as fast as possible, without running, on a premeasured 11 m course. The time taken to walk 5 m, from the 3 m to 8 m mark was recorded and used to calculate maximum walking speed (MWS, in meters/second).

*Functional Reach* test is the difference, in centimeters (cm), between arm's length and maximal forward reach, using a fixed base of support (such as a wall). This test is used to detect balance impairment, changes in balance performance over time, and in the design of modified environments for impaired older persons (Duncan et al., 1990).

*Lower‐Extremity Power* was estimated by assessing maximum vertical jump height according to Bosco et al (Bosco et al., 1983). This test is safe for community‐dwelling older adults, and those with relatively low vertical jump scores had higher mortality rates over the 6‐year follow‐up period (Fujita et al., 1995). Furthermore, as compared to lower‐extremity muscular strength, power is more related to fallrisk (Perry et al., 2007) and overall physical function (Kuh et al., 2005). Participants completed a standard warm‐up consisting of five submaximal jumps, and then three countermovement jumps on a stationary Kistler force plate (Kistler Instruments Corp, Amherst, NY). The “flight‐time” for each jump, determined from the force plate reading, was used to determine the maximum height reached by the subject's center of mass using the Bosco method (Bosco et al., 1983).

### 2.5 Statistical methods

**FIGURE 1:**
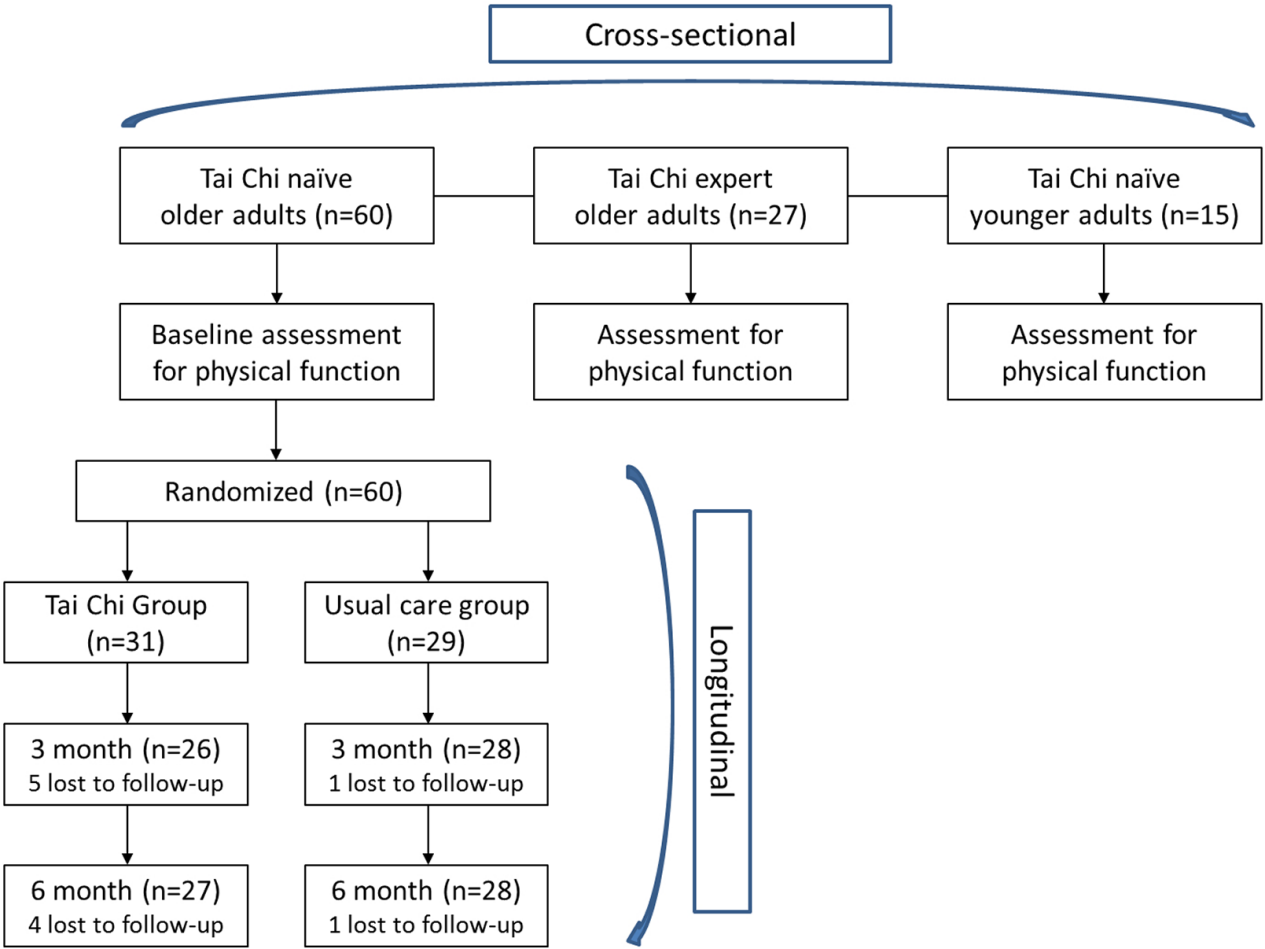
Flow of the study with hybrid design.

Longitudinal analyses for the RCT were performed using SAS statistical software version 9.4 (SAS Institute Inc. Cary, NC). All other statistical analyses were performed using IBM SPSS Statistics version 19 (IBM Corp., Armonk, NY, USA). A *p* value less than 0.05 indicates significant differences. One aim of the study was to compare Tai Chi expert and naïve older adults with the healthy young adults who served as the norm group, allowing for exploration of whether long‐term practice of Tai Chi has an impact on age‐related decline in functional outcomes. Cross‐sectional comparisons were performed among three groups: Tai Chi‐expert older adults, Tai Chi‐naïve older adults, and Tai Chi‐naïve younger adults (Figure 1). Continuous data with non‐normal distributions or violations of the assumption of homogeneity of variance (by Levene's test) were converted by natural log transformation. The results of those variables were reported as geometric means ± standard deviation. Group differences were tested by ANOVA. When significant differences were found among groups, post hoc pair‐wise comparisons were conducted with Bonferroni.

Next, to understand whether Tai Chi can impact age‐related decline in functional outcomes, using baseline data, we examined the associations between age and functional outcomes among Tai Chi experts and among Tai Chi naïve, we then compared the slopes from these regression models. We also explored whether sex modified the association between Tai Chi experts versus Tai Chi naïve and baseline functional measures.

Another aim of the study was to explore, in an RCT, whether a short‐term Tai Chi training could show effects on physical measures in the naïve older adults. A total of 60 Tai Chi‐naive older adults were randomized into the Tai Chi group (*n* = 31) and the wait‐list control group (*n* = 29). During the 6‐month study, five participants from the Tai Chi group, and one from the control group dropped out (Figure 1). Longitudinal comparisons were made using generalized linear models to examine the group, time, and group x time effects of Tai Chi between those randomized to Tai Chi versus a wait‐list control. Generalized linear models adjusted for age, sex, BMI, and baseline activity levels as covariates were also created in adjusted models. Further, to understand the dosage effect, we explored the association between total hours of practice and changes of functional outcomes in those randomized to the Tai Chi group. In visual inspection of the data, we observed an outlier who practiced twice amount of time than others, and we ran analysis both with and without them from the analysis of dosage effect.

## 3 RESULTS

### 3.1 Participant characteristics

Tai Chi experts (*n* = 27) reported an average of 24.6 ± 12 years of Tai Chi training experience (median 20 years, range 10–50 years). On average, experts reported practicing Tai Chi for 8.4 h per week. Approximately equal numbers reported Yang (*n* = 12) and Wu (*n* = 15) style Tai Chi as their primary training systems; however, all experts reported having training experience in other styles of Tai Chi, related internal and external martial arts (e.g., kung fu and bagua), and/or mind–body practices (e.g., yoga and meditation). Sociodemographic characteristics of the Tai Chi experts were well‐balanced with the older Tai Chi‐naïve group with respect to average age and global cognitive status. Compared with naïve older adults, experts included a slightly greater proportion of men and Asians and had a lower BMI and higher levels of physical activity (Table 1).

**TABLE 1:** Demographics and cross‐sectional comparisons.

	Observational groups			Randomized groups and subgroups		
	Tai Chi‐naïve older adults	Tai Chi expert older adults	Tai Chi‐naïve younger adults	Usual care	Tai Chi	Tai Chi compliant
	(*n* = 60)	(*n* = 27)	(*n* = 15)	(*n* = 29)	(*n* = 31)	(*n* = 15)
Age						
Age range (years)	50–79	50–79	25–35	50–79	50–79	50–79
Mean age (years)^a^	64.18 ± 7.68	62.78 ± 7.57	28.73 ± 3.20^b^	64.45 ± 7.42	63.94 ± 8.02	63.47 ± 6.98
Sex *n* (%)						
Men	20 (33.3%)	13 (48.1%)	8 (53.3%)	11 (37.9%)	9 (29%)	5 (33.3%)
Women	40 (66.7%)	14 (51.9%)	7 (46.7%)	18 (62.1%)	22 (71%)	10 (66.7%)
Race *n* (%)						
White	55 (91.7%)	22 (81.5%)	15 (100%)	26 (89.7%)	29 (93.5%)	15 (100%)
African American	3 (5%)	1 (3.7%)	0 (0%)	3 (10.3%)	0 (0%)	0 (0%)
Asian	2 (3.3%)	4 (14.8%)	0 (0%)	0 (0%)	2 (6.5%)	0 (0%)
Ethnicity *n* (%)						
Non‐Hispanic/Non‐Latino	59 (98.3%)	26 (96.3%)	14 (93.3%)	29 (100%)	30 (96.8%)	14 (93.3%)
Hispanic/Latino	1 (1.7%)	1 (3.7%)	1 (6.7%)	0 (0%)	1 (3.2%)	1 (6.7%)
Hypertension *n* (%)	32 (53.3%)	13 (48.1%)	N.A.	17 (58.6%)	15 (48.4%)	6 (40%)
Physical Activity Level^a^ ^,^ ^c^	4.4 ± 2.2	6.0 ± 2.0	N.A.	4.0 ± 2.0	4.0 ± 2.0	4.9 ± 2.2
BMI (kg/m^2^)^a^	26.46 ± 5.46	23.54 ± 2.35	25.43 ± 3.21	26.54 ± 5.83	26.38 ± 5.19	26.81 ± 4.60
Education (years.)^a^	16.7 ± 3.25	18.44 ± 3.34	17.93 ± 2.52	16.19 ± 3.03	17.13 ± 3.41	17.43 ± 2.85
MMSE (out of 30)^a^	29.12 ± 1.01	29.07 ± 1.11	N.A.	29.21 ± 0.82	29.03 ± 1.17	28.93 ± 1.28

^a^ Values provided are mean ± standard deviation.

^b.^ The difference on age compared to naïve younger adults was expected given the study design. No significant difference on age was found between Tai Chi‐naïve older adults and Tai Chi expert older adults.

^c^ Physical Activity Level descriptions: 4 = Run about 1 mile per week OR walk about 1.3 miles per week OR spend about 30 min per week in comparable physical activity. 5 = Run about 1–5 miles per week OR walk 1.3–6 miles per week OR spend 30 to 60 min per week in comparable physical activity. 6 = Run about 6–10 miles per week OR walk 7–13 miles per week OR spend 1–3 h per week in comparable physical activity.

Abbreviations: BMI, body mass index; MMSE, Mini‐Mental State Examination; N.A., not available.

Tai Chi‐naïve older adults subsequently randomized to Tai Chi plus usual care versus usual care alone were comparable at baseline. For all variables, the values for the subset of participants who completed the study were comparable to those in the larger sample, thereby minimizing potential sources of bias in post hoc comparisons between the control and Tai Chi‐compliant groups.

### 3.2 Recruitment and protocol adherence for RCT

Six hundred and seventy‐nine older adults were approached in order to recruit and enroll 60 eligible healthy adults. The majority were excluded due to medical ineligibility (*n* = 214) and limited time availability or interest (*n* = 179). Of those enrolled, 97% (28/29) and 87% (27/31) of individuals in the usual care and Tai Chi groups completed the primary 6‐month follow‐up assessment, respectively. All 60 participants completing baseline assessments were included in intent‐to‐treat analyses. Adherence based on class attendance and self‐reported home practice logs was variable. Fifteen of the 31 (48%) participants in the Tai Chi group were protocol compliant, attending 70% of classes and completing 70% of the required home practice between each study visit. This compliant subgroup had a mean exposure to Tai Chi training of 89.3 h; in comparison, the intent‐to‐treat group had a mean exposure of 60.9 h. Overall satisfaction with Tai Chi programs, assessed on a 1–7 scale (1 is the best score) was high, with a mean ± standard deviation rating of 1.6 ± 1.1 at 3 months and 1.7 ± 1.4 at 6 months.

### 3.3 Long‐term effects of Tai Chi: Cross‐sectional comparisons among the groups

**FIGURE 2:**
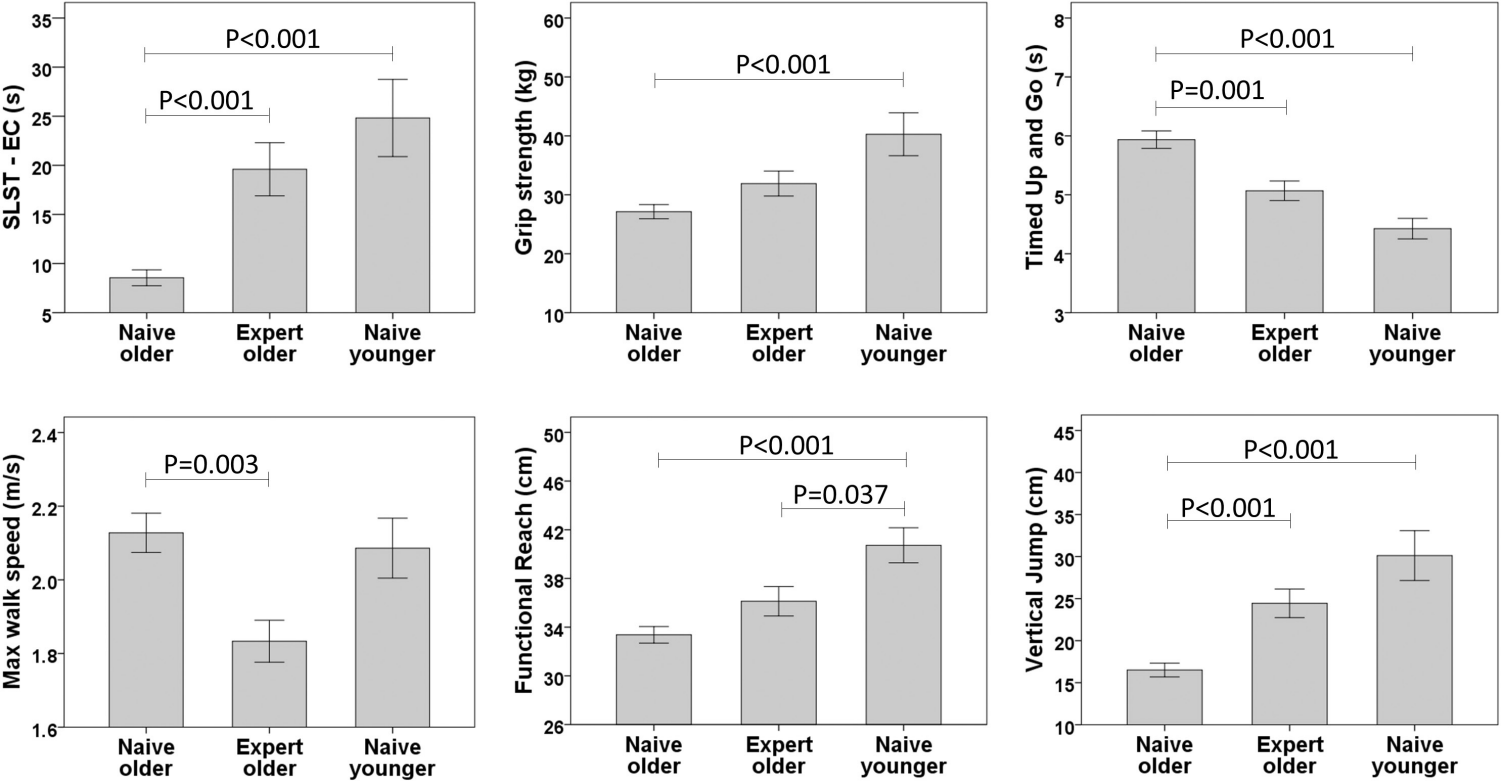
Cross‐sectional group comparisons. Outcomes were continuous variables, presented as mean and error bars for each group. Group comparisons were tested by ANOVA, followed by post hoc tests using Bonferroni for pair‐wise comparisons. When an assumption of Homogeneity of variance was violated (SLST‐EC, grip strength, Timed Up and Go, and vertical jump), raw data were converted by natural log transformation. For Tai Chi‐naïve older adults, all outcome measures were from their baseline visit. All of the global tests were statistically significant, and the *p* values shown in the figures were for the post hoc comparisons. SLST‐EC, single‐legged stance time with eyes closed.

Significant differences were observed among the groups on all six outcome measures (Table 2). Tai Chi experts, as compared to Tai Chi‐naïve older adults, performed better on the SLST‐EC, Timed Up and Go, and vertical jump tests (Figure 2). For all outcomes except max walking speed, Tai Chi‐naïve younger adults exhibited the highest levels of physical function. However, for all but one outcome, absolute differences between Tai Chi‐experts and Tai Chi‐naïve younger adults were smaller than differences between and Tai Chi‐naïve older adults and Tai Chi‐naïve younger adults. The functional reach was the only outcome in which differences between Tai Chi‐experts and Tai Chi‐naïve younger adults reached statistical significance. Interestingly, for max walk speed, Tai Chi experts walked slower than the other two groups (differences compared to Tai Chi‐naïve older adults, but not younger adults, were statistically significant).

**TABLE 2:** Cross‐sectional comparisons of physical function among Tai Chi‐naïve older adults, Tai Chi expert older adults and Tai Chi‐naïve younger adults.

	Tai chi‐naïve older adults (*n* = 60)^a^	Tai chi expert older adults (*n* = 27)^b^	Tai chi‐naïve younger adults (*n* = 15)^c^	*F*	*p*	Pair‐wise comparisons^d^
SLST‐EC (s)^e^	6.64 ± 2.08	15.06 ± 2.18	19.84 ± 2.12	19.29	<0.001	a < b, a < c
Grip strength (kg)^e^	25.67 ± 1.40	30.02 ± 1.44	37.96 ± 1.43	8.22	<0.001	a < c
TUG (s)^e^	5.83 ± 1.21	4.99 ± 1.20	4.38 ± 1.16	17.52	<0.001	a > b, a > c
MWS (m/s)	2.13 ± 0.41	1.83 ± 0.30	2.09 ± 0.32	5.93	0.004	a > b
Functional reach (cm)	33.37 ± 5.28	36.12 ± 6.28	40.72 ± 5.57	10.86	<0.001	a < c, b < c
Vertical jump (cm)^e^	15.24 ± 3.86	22.78 ± 3.78	28.17 ± 3.71	18.17	<0.001	a < b, a < c

*Note*: Continuous variables with normal distributions were reported as mean ± standard deviation. Group differences were tested by ANOVA, followed by post‐hoc test using Bonferroni.

Abbreviations: SLST‐EC, single‐legged stance time with eyes closed; TUG, Timed Up and Go; MWS, maximum walking speed.

^a^ For Tai Chi naïve older adults.

^b^ For Tai chi expert older adults.

^c^ For Tai chi‐naïve younger adults.

^d^ Only significant differences (*p* < 0.05) were presented in the column.

^e^ Assumption of Homogeneity of variance was violated shown in the Levene's test. Therefore, raw data were converted by natural log transformation. Results were reported as geometric means ± standard deviation.

Exploratory analyses evaluated the effect of age and sex on physical function outcomes. We hypothesized that, within the range of 50–79 years of age, Tai Chi would attenuate age‐related decline in physical function. Specifically, we predicted that negative slopes characterizing the relationship between age and function would be less steep in the Tai Chi expert group as compared to the Tai Chi‐naïve group. This was not observed for most outcomes (Figure S1). With respect to sex, while we observed that men exhibited higher levels of physical function than women, there were no significant sex by Tai Chi exposure interactions (Figure S2).

### 3.4 Short‐term effects of Tai Chi: Longitudinal analyses of the randomized control trial

Longitudinal regression models did not reveal significant improvements in any outcome measure when comparing those randomized to Tai Chi to those randomized to usual care (Table 3 and Appendix S1). To explore potential within‐group dosage effects of short‐term Tai Chi practice, we examined the associations between total hours of practice (combination of class and home practice) and changes in functional outcomes from baseline to 6 months. We identified an extreme case (outlier) who practiced twice the amount of time than others. When this individual was included in the model, we observed a significant association between hours of practice and improvement in SLST‐EC, Timed Up and Go, and functional reach. We also observed a statistically nonsignificant trend of improvement in vertical jump. However, after removing this individual from the analyses (Figure S3), while all trends remained the same, no association was statistically significant. Adjustments for age, sex, BMI, and baseline activity level did not change associations in these models.

**TABLE 3:** Longitudinal comparisons of physical function between subjects randomized to 6 months of Tai Chi or to usual care alone control group in the RCT.

Outcomes	Groups	Visit 1 (month 0)	Visit 2 (month 3)	Visit 3 (month 6)	Group	Time	Group*time
SLST‐EC (s)	Tai Chi	9.53 ± 6.67	11.28 ± 11.8	11.54 ± 14.32	0.325	0.422	0.807
	Control	7.52 ± 5.74	8.78 ± 9.27	9.3 ± 12.11			
Grip strength (kg)	Tai Chi	26.44 ± 9.87	27.81 ± 9.96	27.2 ± 9.31	0.722	0.132	0.064
	Control	27.88 ± 8.76	26.96 ± 8.4	28.13 ± 8.68			
TUG (s)	Tai Chi	6.17 ± 1.24	6.02 ± 1.11	5.94 ± 0.99	0.155	0.417	0.614
	Control	5.68 ± 0.99	5.75 ± 0.84	5.68 ± 0.89			
MWS (m/s)	Tai Chi	2.22 ± 0.45	2.14 ± 0.39	2.22 ± 0.42	0.173	0.057	0.473
	Control	2.03 ± 0.35	2.05 ± 0.32	2.12 ± 0.38			
Functional reach (cm)	Tai Chi	31.95 ± 5.46	33.4 ± 5.28	33.12 ± 5.36	0.083	0.596	0.464
	Control	34.87 ± 4.72	34.11 ± 5.92	35.15 ± 5.74			
Vertical jump (cm)	Tai Chi	16.18 ± 6.12	17.22 ± 7.8	17.63 ± 7.49	0.833	0.006	0.725
	Control	16.84 ± 6.65	17.6 ± 7.04	17.48 ± 7.34			

*Note*: Assumption of Homogeneity of variance was not violated shown in the Levene's test. All continuous variables were reported as mean ± standard deviation. Longitudinal comparisons of physical function were conducted using generalized linear models.

Abbreviations: SLST‐EC, single‐legged stance time with eyes closed; TUG, Timed Up and Go; MWS, maximum walking speed.

## 4 DISCUSSION

Findings based on the unique hybrid design employed in this study suggest that long‐term, but not short‐term, Tai Chi training is associated with better outcomes in physical function in healthy and already active adults. In cross‐sectional comparisons, for all outcomes except MWS, older adults with long‐term Tai Chi training (i.e., experts) performed better than age‐matched Tai Chi‐naïve adults. Moreover, Tai Chi experts' functional levels were consistently intermediate between Tai Chi‐naïve older and younger adults. For three of the six outcomes (SLST‐EC, Timed Up and Go, and vertical jump), higher levels of function observed in Tai Chi experts compared to Tai Chi‐naïve older adults were statistically significant and, as described in the discussion below, clinically meaningful. By contrast, findings from the randomized trial component of our hybrid design suggest that short‐term practice of Tai Chi (i.e., 6 months of training) did not result in significant improvement in any measures of physical function in our healthy, active population.

The effectiveness of Tai Chi for fall prevention in older ambulatory adults and better performance on SLST, FR, and TUG have been associated with reduced fall risk, including in prior cross‐sectional studies evaluating Tai Chi. For SLST‐EC, Tai Chi experts were intermediate to Tai Chi‐naïve younger controls and older adults, supporting the idea that long‐term training may be associated with attenuated age‐related decline in this domain of balance. Prior studies have reported that a reduction of 3–4 s of SLST is associated with 10 years of aging (Jonsson et al., 2004). Our findings that Tai Chi experts had an average of more than 15 s of SLST, compared to age‐matched controls, support the positive impact of long‐term Tai Chi exercise on age‐related functional decline in this outcome.

The TUG has been widely used to characterize physical function and mobility. Longer TUG durations are associated with mortality, risk of falls, frailty, and death. As with SLST‐EC, TUG scores in Tai Chi experts were intermediate between Tai Chi‐naïve older and younger adults and statistically better than Tai Chi‐naïve older adults. In healthy older adults aged over 50 years, normative values of mean TUG were reported to be 6–11.8 s (Bohannon, 2006; Nakhostin‐Ansari et al., 2022; Svinøy et al., 2021). In our study, all three exposure groups had better TUG compared to age‐matched published normative values. A recent systematic review reported that a mean difference of as small as 0.63 s (95% CI 0.14–1.12 s) in the time to complete the TUG distinguishes fallers and nonfallers (Schoene et al., 2013). In our study, Tai Chi experts completed the TUG approximately 1 s faster than Tai Chi‐naïve adults. This supports that long‐term Tai Chi training may reduce the risk of falling and more generally is associated with higher levels of function for this outcome.

The functional reach test is a proxy of fall risk in older or disabled subjects. Normative functional reach data from community‐dwelling older adults over 60 years old (*n* = 21 studies) has been reported to be 26.6 cm. For comparable noncommunity frail older adults (*n* = 5 studies), functional reach is 15.4 cm (Rosa et al., 2019). A reach of less than 6 cm is associated with a four times greater risk for falls during the following 6 months, and a score between 15.24 and 25.4 cm indicates a moderate risk for falls, or a twofold greater risk for falls during the following 6 months (Williams et al., 2017). In our study, functional reach even among Tai Chi‐naïve adults was substantially higher (i.e., 33.37 cm) than published normative data, reflecting the high level of health in this population. Nevertheless, Tai Chi experts still exhibited a slightly higher functional reach (2.75 cm) compared to Tai Chi‐naïve adults.

The routine use of grip strength is recommended for identifying older adults at risk of poor health status. Based on normative data from healthy adults in North America, mean grip strength was 22–55 kg in young adults (20–40 years), with an approximately 15–20 kg reduction in older adults (50–80 years) (Dodds et al., 2016). In this study, Tai Chi experts had grip strengths intermediate to Tai Chi‐naïve younger controls and older adults; however, comparisons between individual groups were not statistically significant. Other cross‐sectional studies also support the benefit of Tai Chi for attenuating age‐related decline in grip strength (Stagi et al., 2020; Zheng et al., 2022).

Lower‐extremity strength is correlated with gait speed, gait endurance, and functional balance. In one published study, the mean reported jump height in older adults (age 50–70 years) was 21.4–29.4 cm in females and 29.4–44.3 cm in males (“Vertical Jump Test Normative Data,” 2016). By comparison, in another study with young adults aged 21–30 years, the mean jump height was 55.88 cm for men and 35.56 cm for women (Patterson & Peterson, 2004). In our study, we observed that Tai Chi experts had significantly greater lower‐extremity power compared to Tai Chi‐naive older adults (50% better performance) and only modestly lower power compared to Tai Chi‐naïve younger adults (differences were not significant). These findings suggest that long‐term training may be associated with a higher level of lower‐extremity power and overall function. Parallel findings from a study of middle‐aged and elderly healthy individuals showed that long‐term Tai Chi training (over 5 years) significantly increased jump height (60%), peak knee moment (19.80%), peak ankle moment (8.06%), peak hip power (29.80%), peak knee power (31.23%), and peak ankle power (16.88%) during the takeoff phase of a countermovement jump (Ko et al., 2022).

Interestingly, although the difference on MWS was not statistically significant between older Tai Chi experts and young healthy controls, the Tai Chi experts tended to walk at a slower speed. Tai Chi is a gentle exercise and consists of slow‐motion workouts with strongly focused attention, which may lead to both highly developed control of body motions and more “mindful walking”. Prior studies have reported slower walking following Tai Chi training, including studies in which balance and fall risk were markedly improved. Data collected in this study and reported elsewhere, add context to these findings (Gow et al., 2017; Jor'dan et al., 2018; Ma et al., 2019; Walsh et al., 2015; Wayne et al., 2013, 2014, 2015, 2021). For the two older groups (Tai Chi expert vs. Tai Chi naïve) under dual task (walking while performing serial subtraction) conditions, we observed that gait speed decreased and stride time variability increased, but stride time variability was significantly lower in the Tai Chi‐expert versus Tai Chi‐naïve group. By contrast, gait speed during both undisturbed and dual‐task conditions did not differ between the two groups (Wayne et al., 2015). We also observed that lower extremity muscle co‐contraction (measured by electromyography), an informative clinical marker of mobility health, was lower (i.e., healthier) in Tai Chi experts compared with Tai Chi‐naïve older adults under both quiet and dual‐task walking (Wayne et al., 2021). Collectively, these results suggest that while long‐term Tai Chi training may preserve gait health, its impact may be gait outcome specific. Future studies should examine the association of long‐term Tai Chi training on multiple metrics of gait health, and explore the intriguing finding of reduced speed despite more generally improved overall physical function.

In contrast to the positive impact of long‐term Tai Chi training on physical function, exposure to 6 months of training among the healthy adults recruited for this study had few apparent benefits. This finding was somewhat unexpected, as prior studies of older adults exposed to similar or even shorter periods of training have reported a positive impact on nearly all domains of physical function, including SLST‐EC, FR, TUG, grip strength, lower‐extremity muscle strength, and sometimes MWS. In some studies, such effects were observed even when Tai Chi was compared to an active, alternative exercise control, or standard treatment. One possible explanation for our null finding is the relatively healthy inclusion criteria we employed. When compared to published norms and reference values, our Tai Chi‐naïve participants performed better in several physical function outcomes as mentioned above. This is different from other randomized trials in adults, where specific medical conditions were studied (e.g., high fall risk, Parkinson's disease, and heart failure) and average levels of daily exercise at baseline were more modest. Consequently, it is possible that for this already active population, a more intensive or extended dose of training is needed to impact physical function. Indeed, the exploratory dose–response analyses supported this notion, especially for functional reach and TUG (Figure S3).

In our study, we found significant sex differences on some physical function measures (e.g., grip strength, max walk speed, and vertical jump, Figure S2). However, there were no significant differences on the interaction of sex and Tai Chi practice, indicating that both men and women may equally benefit from Tai Chi. Future studies should further evaluate sex as an effect modifier of the impact of Tai Chi on physical function.

There are a number of strengths to this study. One strength is our hybrid design and our ability to evaluate long‐ and short‐term training of Tai Chi. Our focus on healthy older adults who were already physically active extends our knowledge beyond Tai Chi's effects on clinical populations. Inclusion of younger healthy controls also allows novel comparisons that inform future hypotheses about Tai Chi's potential to attenuate age‐related functional decline. Second, our hybrid included both longitudinal evaluations of short‐term Tai Chi training in an RCT and cross‐sectional comparisons among Tai Chi experts, Tai Chi naïve older adults, and younger healthy controls. Importantly, our hybrid design utilized the same outcomes assessment protocol in the randomized and observational substudies, eliminating heterogeneity in outcomes, laboratory equipment and testing environment, assessment protocol, and research staff. In addition, our pragmatic RCT enabled us to understand the effects of Tai Chi for older adults learning in real‐world community schools/centers (instead of following a designated research instructor at a makeshift hospital exercise environment), with results generalizable across many styles of Tai Chi. Therefore, the results of our study substantially extend new knowledge to the existing publications.

Our study also has important limitations. As an exploratory study of secondary outcomes, sample sizes in both the cross‐sectional and randomized studies were small, and these analyses may have been underpowered to detect differences among the groups. As with any cross‐sectional study, group comparisons may have been confounded by differences between groups other than Tai Chi exposure. Of note, the majority of Tai Chi experts reported cross‐training in other martial arts, thus associations between Tai Chi training and functional abilities should be interpreted cautiously. Future studies might limit inclusion to Tai Chi experts with more narrow training histories. Similarly, Tai Chi‐naïve participants also reported high levels of physical activity (e.g., walking and fitness training) in addition to their 6‐month exposure to Tai Chi. Future, more adequately powered studies should attempt to include data on the types and amounts of extracurricular physical activity as covariates in statistical models. In our cross‐sectional analyses, although we considered multiple potential confounders, observed between‐group differences may have been impacted by additional factors which we did not assess. In our randomized trial, both the short duration of exposure (6 months) and the very high levels of health of participants at baseline may have contributed to the null effect of Tai Chi's short‐term effect on the improvement of physical function, especially given that our older population was already physically active. The short‐term effects of Tai Chi on physical function in active healthy adults will need to be confirmed in larger, adequately powered studies. Finally, as noted above, our pragmatic approach included studying the effects of Tai Chi across many styles of Tai Chi. While this affords generalizability, our study was not designed to systematically assess potential benefits unique to different Tai Chi styles or methods of instruction. Future studies should explore potential differences in effectiveness between styles in well‐designed randomized and cross‐sectional trials.

## 5 CONCLUSIONS

Long‐term Tai Chi training is associated with higher levels of physical function in older adults, suggesting a potential preventative healthy aging effect. By contrast, short‐term practice of Tai Chi (i.e., 6 months of training) may not lead to significant improvements in physical function in a healthy, active population of older adults. The impact of short‐term training in healthy older adults is less clear. Both potential longer‐term preventive effects and shorter‐term restorative effects warrant further research with rigorous, adequately powered controlled clinical trials.

## AUTHOR CONTRIBUTIONS

PMW conceived and designed the study; YM and BJG organized original raw data; YM and PMW carried out the analyses and drafted the manuscript; YM, PMW, and PMR carried out the performed the statistical analyses; RS, PMR, JMH, LAL, and BM reviewed and helped to revise the manuscript. All authors have read and approved the final version of the manuscript, and agree with the order of presentation of the authors.

## CONFLICT OF INTEREST STATEMENT

Peter Wayne is the founder and sole owner of the Tree of Life Tai Chi Center. Peter Wayne's interests were reviewed and managed by the Brigham and Women's Hospital and Partner's HealthCare in accordance with their conflict of interest policies. This does not alter our adherence to the journal's policies on sharing data and materials. No other authors have any potential conflicts to disclose.

## Supporting information


Figures S1–S3

Appendix S1

## Data Availability

The data that support the findings of this study are available from the corresponding author upon reasonable request.
