# Supplementary material for: Long‐term Tai Chi practice in older adults is associated with “younger” functional abilities
Source: Aging Cell. 2023 Oct 31;23(1):e14023. doi: 10.1111/acel.14023 (PMC10776109; doi:10.1111/acel.14023)
Supplement: Supplementary file 1 — Figures S1–S3 [file ACEL-23-e14023-s001.docx]

**Supplementary Figure 1**: Physical function with ageing.


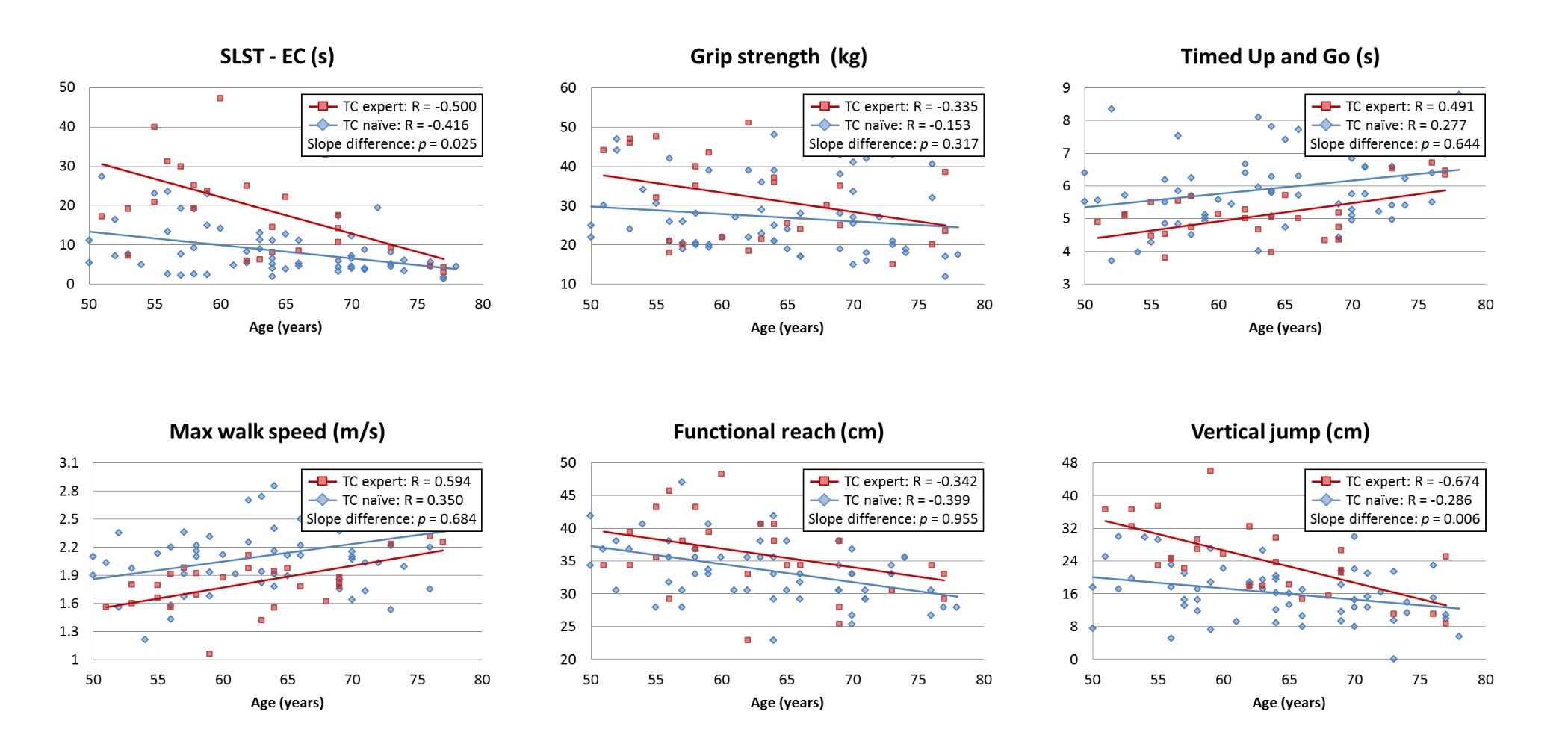


For Tai Chi-naïve older adults, all outcome measures were from their baseline visit. Differences in the slopes were based on the interaction term of age and Tai Chi exposure using linear regression models.

Abbreviations: SLST-EC, single-legged stance time with eyes closed.

**Supplementary Figure 2**: Cross-sectional group comparisons by sex.


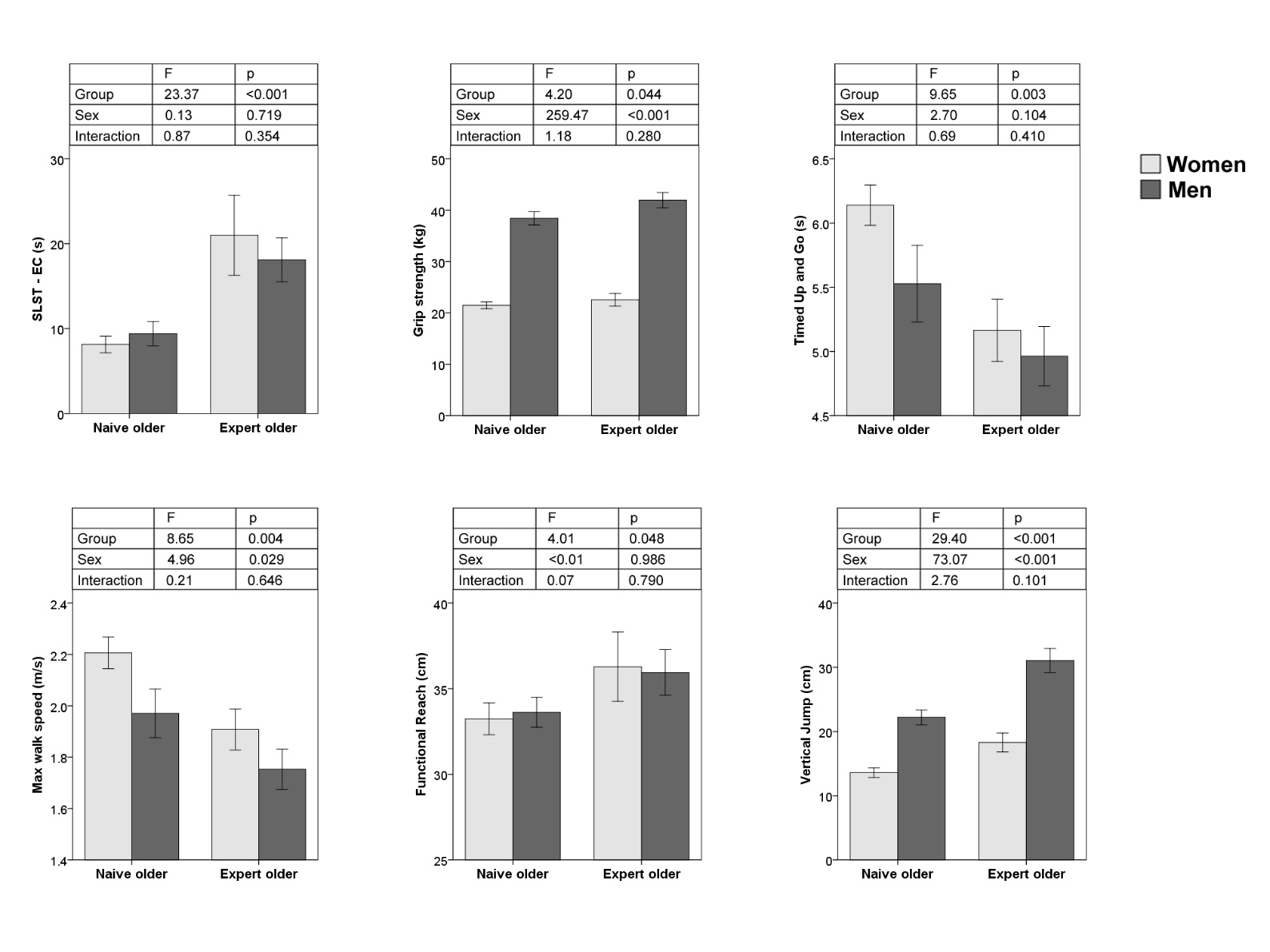


Outcomes were continuous variables, presented as mean and error bars for each group. Group comparisons were tested by linear regression model. The effects of group, sex and group*gender interaction were reported above each figure. For Tai Chi-naïve older adults, all outcome measures were from their baseline visit. Abbreviations: SLST-EC, single-legged stance time with eyes closed.

**Supplementary Figure 3**. Dosage effect of short-term Tai Chi practice.


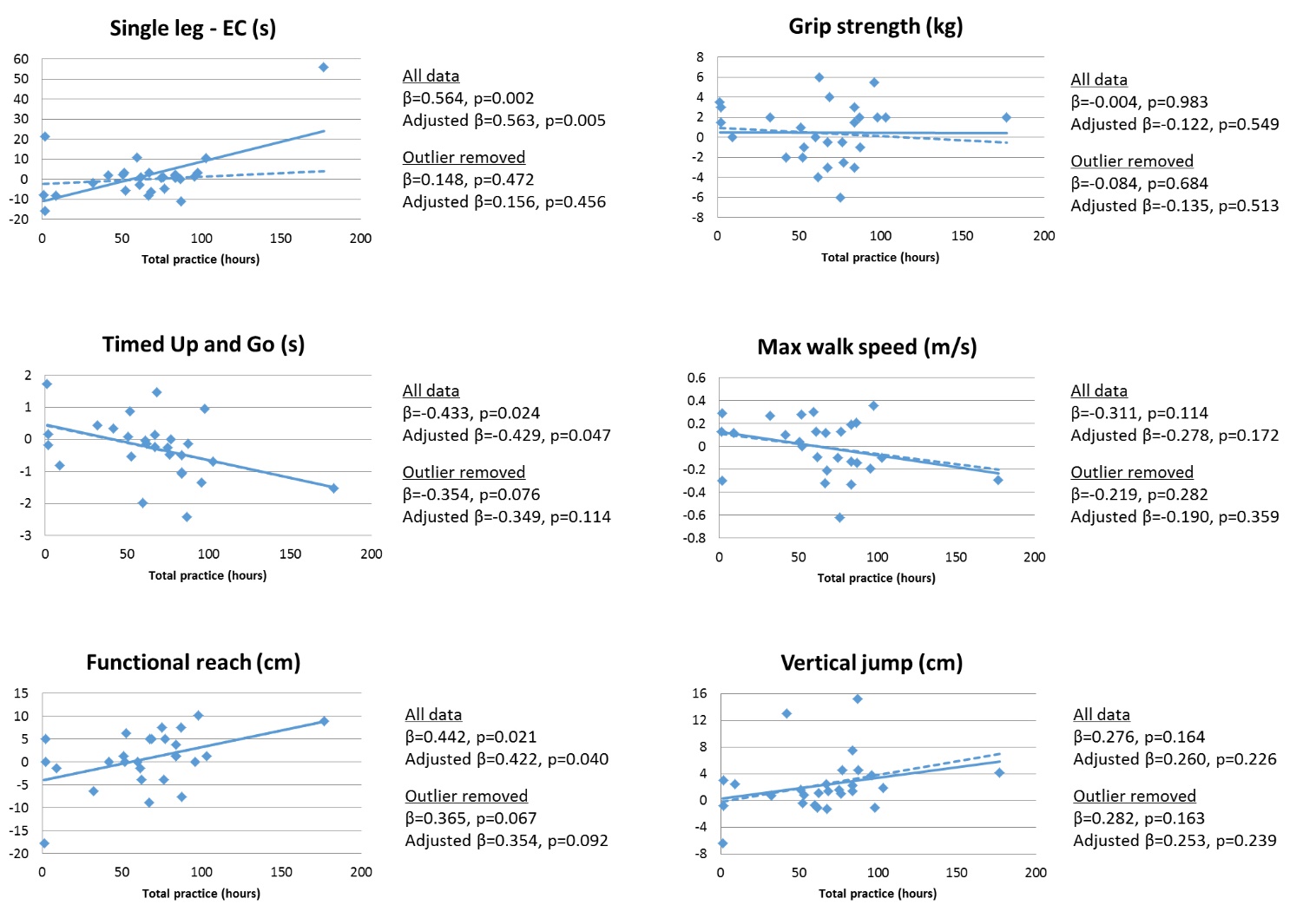


Total practice time was the combination of the total hours for class and home practice. Y-axis shows the changes in functional outcomes from the baseline to the 6th month. Solid lines indicate slopes for all data, and dash lines show slopes after the outlier was removed. β and *p* were based on un-adjusted linear regression models; β’ and *p’* were based on linear regression models that adjusted for age, sex, BMI, and activity.

Abbreviations: SLST-EC, single-legged stance time with eyes closed.
