# Supplementary material for: Long‐term Tai Chi practice in older adults is associated with “younger” functional abilities
Source: Aging Cell. 2023 Oct 31;23(1):e14023. doi: 10.1111/acel.14023 (PMC10776109; doi:10.1111/acel.14023)
Supplement: Supplementary file 2 — Appendix S1 [file ACEL-23-e14023-s002.doc]

| **Model Information** | |
| --- | --- |
| **Data Set** | WORK.SLST |
| **Dependent Variable** | SLST |
| **Covariance Structure** | Unstructured |
| **Subject Effect** | id |
| **Estimation Method** | REML |
| **Residual Variance Method** | None |
| **Fixed Effects SE Method** | Model-Based |
| **Degrees of Freedom Method** | Between-Within |

| **Solution for Fixed Effects** | | | | | | | |
| --- | --- | --- | --- | --- | --- | --- | --- |
| **Effect** | **group** | **time** | **Estimate** | **Standard Error** | **DF** | **t Value** | **Pr > |t|** |
| **Intercept** |  |  | 7.5159 | 1.1585 | 58 | 6.49 | <.0001 |
| **group** | 1 |  | 2.0132 | 1.6118 | 58 | 1.25 | 0.2167 |
| **time** |  | 2 | 1.1863 | 1.7313 | 58 | 0.69 | 0.4959 |
| **time** |  | 3 | 1.6894 | 2.2436 | 58 | 0.75 | 0.4545 |
| **group*time** | 1 | 2 | 0.8522 | 2.4436 | 58 | 0.35 | 0.7286 |
| **group*time** | 1 | 3 | 0.03424 | 3.1594 | 58 | 0.01 | 0.9914 |

| **Type 3 Tests of Fixed Effects** | | | | | | |
| --- | --- | --- | --- | --- | --- | --- |
| **Effect** | **Num DF** | **Den DF** | **Chi-Square** | **F Value** | **Pr > ChiSq** | **Pr > F** |
| **group** | 1 | 58 | 0.98 | 0.98 | 0.3211 | 0.3252 |
| **time** | 2 | 58 | 1.75 | 0.88 | 0.4167 | 0.4221 |
| **group*time** | 2 | 58 | 0.43 | 0.22 | 0.8060 | 0.8067 |

| **Model Information** | |
| --- | --- |
| **Data Set** | WORK.SLST |
| **Dependent Variable** | SLST |
| **Covariance Structure** | Unstructured |
| **Subject Effect** | id |
| **Estimation Method** | REML |
| **Residual Variance Method** | None |
| **Fixed Effects SE Method** | Model-Based |
| **Degrees of Freedom Method** | Between-Within |

| **Solution for Fixed Effects** | | | | | | | |
| --- | --- | --- | --- | --- | --- | --- | --- |
| **Effect** | **group** | **time** | **Estimate** | **Standard Error** | **DF** | **t Value** | **Pr > |t|** |
| **Intercept** |  |  | 32.1777 | 8.8839 | 54 | 3.62 | 0.0006 |
| **age** |  |  | -0.3316 | 0.1001 | 54 | -3.31 | 0.0017 |
| **male** |  |  | 1.3420 | 1.6400 | 54 | 0.82 | 0.4168 |
| **BMI** |  |  | -0.1499 | 0.1538 | 54 | -0.97 | 0.3342 |
| **Activity** |  |  | 0.02754 | 0.3677 | 54 | 0.07 | 0.9406 |
| **group** | 1 |  | 1.8757 | 1.5123 | 54 | 1.24 | 0.2202 |
| **time** |  | 2 | 1.2097 | 1.7269 | 54 | 0.70 | 0.4866 |
| **time** |  | 3 | 1.7039 | 2.2433 | 54 | 0.76 | 0.4508 |
| **group*time** | 1 | 2 | 0.9100 | 2.4379 | 54 | 0.37 | 0.7104 |
| **group*time** | 1 | 3 | 0.06040 | 3.1592 | 54 | 0.02 | 0.9848 |

| **Type 3 Tests of Fixed Effects** | | | | | | |
| --- | --- | --- | --- | --- | --- | --- |
| **Effect** | **Num DF** | **Den DF** | **Chi-Square** | **F Value** | **Pr > ChiSq** | **Pr > F** |
| **age** | 1 | 54 | 10.97 | 10.97 | 0.0009 | 0.0017 |
| **male** | 1 | 54 | 0.67 | 0.67 | 0.4132 | 0.4168 |
| **BMI** | 1 | 54 | 0.95 | 0.95 | 0.3299 | 0.3342 |
| **Activity** | 1 | 54 | 0.01 | 0.01 | 0.9403 | 0.9406 |
| **group** | 1 | 54 | 0.93 | 0.93 | 0.3336 | 0.3379 |
| **time** | 2 | 54 | 1.88 | 0.94 | 0.3900 | 0.3963 |
| **group*time** | 2 | 54 | 0.47 | 0.24 | 0.7887 | 0.7895 |

| **Model Information** | |
| --- | --- |
| **Data Set** | WORK.GRIPS |
| **Dependent Variable** | GripS |
| **Covariance Structure** | Unstructured |
| **Subject Effect** | id |
| **Estimation Method** | REML |
| **Residual Variance Method** | None |
| **Fixed Effects SE Method** | Model-Based |
| **Degrees of Freedom Method** | Between-Within |

| **Solution for Fixed Effects** | | | | | | | |
| --- | --- | --- | --- | --- | --- | --- | --- |
| **Effect** | **group** | **time** | **Estimate** | **Standard Error** | **DF** | **t Value** | **Pr > |t|** |
| **Intercept** |  |  | 27.8793 | 1.7358 | 58 | 16.06 | <.0001 |
| **group** | 1 |  | -1.4438 | 2.4148 | 58 | -0.60 | 0.5522 |
| **time** |  | 2 | -0.7124 | 0.6011 | 58 | -1.19 | 0.2408 |
| **time** |  | 3 | 0.4514 | 0.4381 | 58 | 1.03 | 0.3072 |
| **group*time** | 1 | 2 | 1.6588 | 0.8550 | 58 | 1.94 | 0.0572 |
| **group*time** | 1 | 3 | 0.1476 | 0.6198 | 58 | 0.24 | 0.8126 |

| **Type 3 Tests of Fixed Effects** | | | | | | |
| --- | --- | --- | --- | --- | --- | --- |
| **Effect** | **Num DF** | **Den DF** | **Chi-Square** | **F Value** | **Pr > ChiSq** | **Pr > F** |
| **group** | 1 | 58 | 0.13 | 0.13 | 0.7202 | 0.7215 |
| **time** | 2 | 58 | 4.20 | 2.10 | 0.1226 | 0.1318 |
| **group*time** | 2 | 58 | 5.78 | 2.89 | 0.0556 | 0.0637 |

| **Model Information** | |
| --- | --- |
| **Data Set** | WORK.GRIPS |
| **Dependent Variable** | GripS |
| **Covariance Structure** | Unstructured |
| **Subject Effect** | id |
| **Estimation Method** | REML |
| **Residual Variance Method** | None |
| **Fixed Effects SE Method** | Model-Based |
| **Degrees of Freedom Method** | Between-Within |

| **Solution for Fixed Effects** | | | | | | | |
| --- | --- | --- | --- | --- | --- | --- | --- |
| **Effect** | **group** | **time** | **Estimate** | **Standard Error** | **DF** | **t Value** | **Pr > |t|** |
| **Intercept** |  |  | 27.8074 | 6.7650 | 54 | 4.11 | 0.0001 |
| **age** |  |  | -0.1692 | 0.07615 | 54 | -2.22 | 0.0305 |
| **male** |  |  | 16.6222 | 1.2423 | 54 | 13.38 | <.0001 |
| **BMI** |  |  | 0.1605 | 0.1165 | 54 | 1.38 | 0.1741 |
| **Activity** |  |  | 0.1124 | 0.2786 | 54 | 0.40 | 0.6882 |
| **group** | 1 |  | 0.002599 | 1.1967 | 54 | 0.00 | 0.9983 |
| **time** |  | 2 | -0.6654 | 0.6012 | 54 | -1.11 | 0.2733 |
| **time** |  | 3 | 0.4981 | 0.4366 | 54 | 1.14 | 0.2589 |
| **group*time** | 1 | 2 | 1.6069 | 0.8553 | 54 | 1.88 | 0.0657 |
| **group*time** | 1 | 3 | 0.1132 | 0.6171 | 54 | 0.18 | 0.8552 |

| **Type 3 Tests of Fixed Effects** | | | | | | |
| --- | --- | --- | --- | --- | --- | --- |
| **Effect** | **Num DF** | **Den DF** | **Chi-Square** | **F Value** | **Pr > ChiSq** | **Pr > F** |
| **age** | 1 | 54 | 4.93 | 4.93 | 0.0263 | 0.0305 |
| **male** | 1 | 54 | 179.02 | 179.02 | <.0001 | <.0001 |
| **BMI** | 1 | 54 | 1.90 | 1.90 | 0.1684 | 0.1741 |
| **Activity** | 1 | 54 | 0.16 | 0.16 | 0.6866 | 0.6882 |
| **group** | 1 | 54 | 0.25 | 0.25 | 0.6186 | 0.6206 |
| **time** | 2 | 54 | 4.61 | 2.30 | 0.1000 | 0.1097 |
| **group*time** | 2 | 54 | 5.58 | 2.79 | 0.0614 | 0.0703 |

| **Model Information** | |
| --- | --- |
| **Data Set** | WORK.TUG |
| **Dependent Variable** | TUG |
| **Covariance Structure** | Unstructured |
| **Subject Effect** | id |
| **Estimation Method** | REML |
| **Residual Variance Method** | None |
| **Fixed Effects SE Method** | Model-Based |
| **Degrees of Freedom Method** | Between-Within |

| **Solution for Fixed Effects** | | | | | | | |
| --- | --- | --- | --- | --- | --- | --- | --- |
| **Effect** | **group** | **time** | **Estimate** | **Standard Error** | **DF** | **t Value** | **Pr > |t|** |
| **Intercept** |  |  | 5.6838 | 0.2092 | 58 | 27.16 | <.0001 |
| **group** | 1 |  | 0.4872 | 0.2911 | 58 | 1.67 | 0.0996 |
| **time** |  | 2 | 0.03071 | 0.1553 | 58 | 0.20 | 0.8439 |
| **time** |  | 3 | -0.03073 | 0.1829 | 58 | -0.17 | 0.8671 |
| **group*time** | 1 | 2 | -0.1926 | 0.2195 | 58 | -0.88 | 0.3839 |
| **group*time** | 1 | 3 | -0.2505 | 0.2573 | 58 | -0.97 | 0.3342 |

| **Type 3 Tests of Fixed Effects** | | | | | | |
| --- | --- | --- | --- | --- | --- | --- |
| **Effect** | **Num DF** | **Den DF** | **Chi-Square** | **F Value** | **Pr > ChiSq** | **Pr > F** |
| **group** | 1 | 58 | 2.08 | 2.08 | 0.1496 | 0.1549 |
| **time** | 2 | 58 | 1.78 | 0.89 | 0.4111 | 0.4166 |
| **group*time** | 2 | 58 | 0.98 | 0.49 | 0.6114 | 0.6139 |

| **Model Information** | |
| --- | --- |
| **Data Set** | WORK.TUG |
| **Dependent Variable** | TUG |
| **Covariance Structure** | Unstructured |
| **Subject Effect** | id |
| **Estimation Method** | REML |
| **Residual Variance Method** | None |
| **Fixed Effects SE Method** | Model-Based |
| **Degrees of Freedom Method** | Between-Within |

| **Solution for Fixed Effects** | | | | | | | |
| --- | --- | --- | --- | --- | --- | --- | --- |
| **Effect** | **group** | **time** | **Estimate** | **Standard Error** | **DF** | **t Value** | **Pr > |t|** |
| **Intercept** |  |  | 1.5242 | 1.3094 | 54 | 1.16 | 0.2495 |
| **age** |  |  | 0.04416 | 0.01464 | 54 | 3.02 | 0.0039 |
| **male** |  |  | -0.4112 | 0.2362 | 54 | -1.74 | 0.0874 |
| **BMI** |  |  | 0.05293 | 0.02217 | 54 | 2.39 | 0.0205 |
| **Activity** |  |  | 0.02050 | 0.05308 | 54 | 0.39 | 0.7008 |
| **group** | 1 |  | 0.4956 | 0.2792 | 54 | 1.77 | 0.0815 |
| **time** |  | 2 | 0.01994 | 0.1542 | 54 | 0.13 | 0.8976 |
| **time** |  | 3 | -0.04363 | 0.1807 | 54 | -0.24 | 0.8101 |
| **group*time** | 1 | 2 | -0.1983 | 0.2180 | 54 | -0.91 | 0.3669 |
| **group*time** | 1 | 3 | -0.2581 | 0.2541 | 54 | -1.02 | 0.3143 |

| **Type 3 Tests of Fixed Effects** | | | | | | |
| --- | --- | --- | --- | --- | --- | --- |
| **Effect** | **Num DF** | **Den DF** | **Chi-Square** | **F Value** | **Pr > ChiSq** | **Pr > F** |
| **age** | 1 | 54 | 9.09 | 9.09 | 0.0026 | 0.0039 |
| **male** | 1 | 54 | 3.03 | 3.03 | 0.0817 | 0.0874 |
| **BMI** | 1 | 54 | 5.70 | 5.70 | 0.0170 | 0.0205 |
| **Activity** | 1 | 54 | 0.15 | 0.15 | 0.6993 | 0.7008 |
| **group** | 1 | 54 | 2.45 | 2.45 | 0.1176 | 0.1234 |
| **time** | 2 | 54 | 2.12 | 1.06 | 0.3471 | 0.3542 |
| **group*time** | 2 | 54 | 1.07 | 0.53 | 0.5860 | 0.5890 |

| **Model Information** | |
| --- | --- |
| **Data Set** | WORK.MAXW |
| **Dependent Variable** | MaxW |
| **Covariance Structure** | Unstructured |
| **Subject Effect** | id |
| **Estimation Method** | REML |
| **Residual Variance Method** | None |
| **Fixed Effects SE Method** | Model-Based |
| **Degrees of Freedom Method** | Between-Within |

| **Solution for Fixed Effects** | | | | | | | |
| --- | --- | --- | --- | --- | --- | --- | --- |
| **Effect** | **group** | **time** | **Estimate** | **Standard Error** | **DF** | **t Value** | **Pr > |t|** |
| **Intercept** |  |  | 2.0300 | 0.07516 | 58 | 27.01 | <.0001 |
| **group** | 1 |  | 0.1887 | 0.1046 | 58 | 1.80 | 0.0763 |
| **time** |  | 2 | -0.00056 | 0.05422 | 58 | -0.01 | 0.9918 |
| **time** |  | 3 | 0.06406 | 0.05407 | 58 | 1.18 | 0.2409 |
| **group*time** | 1 | 2 | -0.09330 | 0.07672 | 58 | -1.22 | 0.2288 |
| **group*time** | 1 | 3 | -0.07485 | 0.07645 | 58 | -0.98 | 0.3316 |

| **Type 3 Tests of Fixed Effects** | | | | | | |
| --- | --- | --- | --- | --- | --- | --- |
| **Effect** | **Num DF** | **Den DF** | **Chi-Square** | **F Value** | **Pr > ChiSq** | **Pr > F** |
| **group** | 1 | 58 | 1.91 | 1.91 | 0.1675 | 0.1728 |
| **time** | 2 | 58 | 6.01 | 3.00 | 0.0496 | 0.0574 |
| **group*time** | 2 | 58 | 1.52 | 0.76 | 0.4680 | 0.4726 |

| **Model Information** | |
| --- | --- |
| **Data Set** | WORK.MAXW |
| **Dependent Variable** | MaxW |
| **Covariance Structure** | Unstructured |
| **Subject Effect** | id |
| **Estimation Method** | REML |
| **Residual Variance Method** | None |
| **Fixed Effects SE Method** | Model-Based |
| **Degrees of Freedom Method** | Between-Within |

| **Solution for Fixed Effects** | | | | | | | |
| --- | --- | --- | --- | --- | --- | --- | --- |
| **Effect** | **group** | **time** | **Estimate** | **Standard Error** | **DF** | **t Value** | **Pr > |t|** |
| **Intercept** |  |  | 0.5501 | 0.4712 | 54 | 1.17 | 0.2482 |
| **age** |  |  | 0.01987 | 0.005274 | 54 | 3.77 | 0.0004 |
| **male** |  |  | -0.2897 | 0.08566 | 54 | -3.38 | 0.0013 |
| **BMI** |  |  | 0.01111 | 0.008064 | 54 | 1.38 | 0.1741 |
| **Activity** |  |  | 0.004471 | 0.01926 | 54 | 0.23 | 0.8173 |
| **group** | 1 |  | 0.1778 | 0.09707 | 54 | 1.83 | 0.0726 |
| **time** |  | 2 | -0.00426 | 0.05331 | 54 | -0.08 | 0.9365 |
| **time** |  | 3 | 0.06413 | 0.05458 | 54 | 1.18 | 0.2451 |
| **group*time** | 1 | 2 | -0.09542 | 0.07536 | 54 | -1.27 | 0.2109 |
| **group*time** | 1 | 3 | -0.07789 | 0.07709 | 54 | -1.01 | 0.3168 |

| **Type 3 Tests of Fixed Effects** | | | | | | |
| --- | --- | --- | --- | --- | --- | --- |
| **Effect** | **Num DF** | **Den DF** | **Chi-Square** | **F Value** | **Pr > ChiSq** | **Pr > F** |
| **age** | 1 | 54 | 14.20 | 14.20 | 0.0002 | 0.0004 |
| **male** | 1 | 54 | 11.44 | 11.44 | 0.0007 | 0.0013 |
| **BMI** | 1 | 54 | 1.90 | 1.90 | 0.1684 | 0.1741 |
| **Activity** | 1 | 54 | 0.05 | 0.05 | 0.8164 | 0.8173 |
| **group** | 1 | 54 | 2.20 | 2.20 | 0.1380 | 0.1438 |
| **time** | 2 | 54 | 6.89 | 3.45 | 0.0318 | 0.0390 |
| **group*time** | 2 | 54 | 1.64 | 0.82 | 0.4412 | 0.4466 |

| **Model Information** | |
| --- | --- |
| **Data Set** | WORK.FUNCR |
| **Dependent Variable** | FuncR |
| **Covariance Structure** | Unstructured |
| **Subject Effect** | id |
| **Estimation Method** | REML |
| **Residual Variance Method** | None |
| **Fixed Effects SE Method** | Model-Based |
| **Degrees of Freedom Method** | Between-Within |

| **Solution for Fixed Effects** | | | | | | | |
| --- | --- | --- | --- | --- | --- | --- | --- |
| **Effect** | **group** | **time** | **Estimate** | **Standard Error** | **DF** | **t Value** | **Pr > |t|** |
| **Intercept** |  |  | 13.7328 | 0.3742 | 58 | 36.70 | <.0001 |
| **group** | 1 |  | -1.1521 | 0.5206 | 58 | -2.21 | 0.0308 |
| **time** |  | 2 | -0.2649 | 0.4402 | 58 | -0.60 | 0.5496 |
| **time** |  | 3 | 0.1527 | 0.4080 | 58 | 0.37 | 0.7097 |
| **group*time** | 1 | 2 | 0.6843 | 0.6241 | 58 | 1.10 | 0.2774 |
| **group*time** | 1 | 3 | 0.2010 | 0.5763 | 58 | 0.35 | 0.7286 |

| **Type 3 Tests of Fixed Effects** | | | | | | |
| --- | --- | --- | --- | --- | --- | --- |
| **Effect** | **Num DF** | **Den DF** | **Chi-Square** | **F Value** | **Pr > ChiSq** | **Pr > F** |
| **group** | 1 | 58 | 3.10 | 3.10 | 0.0781 | 0.0834 |
| **time** | 2 | 58 | 1.04 | 0.52 | 0.5931 | 0.5958 |
| **group*time** | 2 | 58 | 1.56 | 0.78 | 0.4589 | 0.4636 |

| **Model Information** | |
| --- | --- |
| **Data Set** | WORK.FUNCR |
| **Dependent Variable** | FuncR |
| **Covariance Structure** | Unstructured |
| **Subject Effect** | id |
| **Estimation Method** | REML |
| **Residual Variance Method** | None |
| **Fixed Effects SE Method** | Model-Based |
| **Degrees of Freedom Method** | Between-Within |

| **Solution for Fixed Effects** | | | | | | | |
| --- | --- | --- | --- | --- | --- | --- | --- |
| **Effect** | **group** | **time** | **Estimate** | **Standard Error** | **DF** | **t Value** | **Pr > |t|** |
| **Intercept** |  |  | 21.3884 | 2.6175 | 54 | 8.17 | <.0001 |
| **age** |  |  | -0.1058 | 0.02941 | 54 | -3.60 | 0.0007 |
| **male** |  |  | 0.1641 | 0.4787 | 54 | 0.34 | 0.7330 |
| **BMI** |  |  | -0.02037 | 0.04491 | 54 | -0.45 | 0.6520 |
| **Activity** |  |  | -0.08607 | 0.1075 | 54 | -0.80 | 0.4267 |
| **group** | 1 |  | -1.1789 | 0.4883 | 54 | -2.41 | 0.0192 |
| **time** |  | 2 | -0.2449 | 0.4398 | 54 | -0.56 | 0.5799 |
| **time** |  | 3 | 0.1670 | 0.4077 | 54 | 0.41 | 0.6837 |
| **group*time** | 1 | 2 | 0.6995 | 0.6239 | 54 | 1.12 | 0.2672 |
| **group*time** | 1 | 3 | 0.2344 | 0.5756 | 54 | 0.41 | 0.6855 |

| **Type 3 Tests of Fixed Effects** | | | | | | |
| --- | --- | --- | --- | --- | --- | --- |
| **Effect** | **Num DF** | **Den DF** | **Chi-Square** | **F Value** | **Pr > ChiSq** | **Pr > F** |
| **age** | 1 | 54 | 12.95 | 12.95 | 0.0003 | 0.0007 |
| **male** | 1 | 54 | 0.12 | 0.12 | 0.7317 | 0.7330 |
| **BMI** | 1 | 54 | 0.21 | 0.21 | 0.6502 | 0.6520 |
| **Activity** | 1 | 54 | 0.64 | 0.64 | 0.4232 | 0.4267 |
| **group** | 1 | 54 | 3.50 | 3.50 | 0.0614 | 0.0668 |
| **time** | 2 | 54 | 1.23 | 0.62 | 0.5401 | 0.5439 |
| **group*time** | 2 | 54 | 1.55 | 0.77 | 0.4611 | 0.4661 |

| **Model Information** | |
| --- | --- |
| **Data Set** | WORK.VERTJ |
| **Dependent Variable** | VertJ |
| **Covariance Structure** | Unstructured |
| **Subject Effect** | id |
| **Estimation Method** | REML |
| **Residual Variance Method** | None |
| **Fixed Effects SE Method** | Model-Based |
| **Degrees of Freedom Method** | Between-Within |

| **Solution for Fixed Effects** | | | | | | | |
| --- | --- | --- | --- | --- | --- | --- | --- |
| **Effect** | **group** | **time** | **Estimate** | **Standard Error** | **DF** | **t Value** | **Pr > |t|** |
| **Intercept** |  |  | 6.6334 | 0.4658 | 58 | 14.24 | <.0001 |
| **group** | 1 |  | -0.3230 | 0.6488 | 58 | -0.50 | 0.6205 |
| **time** |  | 2 | 0.2500 | 0.2314 | 58 | 1.08 | 0.2844 |
| **time** |  | 3 | 0.4426 | 0.2458 | 58 | 1.80 | 0.0770 |
| **group*time** | 1 | 2 | 0.2564 | 0.3273 | 58 | 0.78 | 0.4366 |
| **group*time** | 1 | 3 | 0.2594 | 0.3484 | 58 | 0.74 | 0.4596 |

| **Type 3 Tests of Fixed Effects** | | | | | | |
| --- | --- | --- | --- | --- | --- | --- |
| **Effect** | **Num DF** | **Den DF** | **Chi-Square** | **F Value** | **Pr > ChiSq** | **Pr > F** |
| **group** | 1 | 58 | 0.04 | 0.04 | 0.8321 | 0.8328 |
| **time** | 2 | 58 | 11.23 | 5.61 | 0.0036 | 0.0059 |
| **group*time** | 2 | 58 | 0.65 | 0.32 | 0.7240 | 0.7253 |

| **Model Information** | |
| --- | --- |
| **Data Set** | WORK.VERTJ |
| **Dependent Variable** | VertJ |
| **Covariance Structure** | Unstructured |
| **Subject Effect** | id |
| **Estimation Method** | REML |
| **Residual Variance Method** | None |
| **Fixed Effects SE Method** | Model-Based |
| **Degrees of Freedom Method** | Between-Within |

| **Solution for Fixed Effects** | | | | | | | |
| --- | --- | --- | --- | --- | --- | --- | --- |
| **Effect** | **group** | **time** | **Estimate** | **Standard Error** | **DF** | **t Value** | **Pr > |t|** |
| **Intercept** |  |  | 17.1527 | 2.3726 | 54 | 7.23 | <.0001 |
| **age** |  |  | -0.1210 | 0.02674 | 54 | -4.52 | <.0001 |
| **male** |  |  | 3.3927 | 0.4361 | 54 | 7.78 | <.0001 |
| **BMI** |  |  | -0.1673 | 0.04089 | 54 | -4.09 | 0.0001 |
| **Activity** |  |  | 0.08544 | 0.09809 | 54 | 0.87 | 0.3875 |
| **group** | 1 |  | -0.2152 | 0.4215 | 54 | -0.51 | 0.6117 |
| **time** |  | 2 | 0.2349 | 0.2306 | 54 | 1.02 | 0.3129 |
| **time** |  | 3 | 0.4190 | 0.2456 | 54 | 1.71 | 0.0938 |
| **group*time** | 1 | 2 | 0.2815 | 0.3263 | 54 | 0.86 | 0.3921 |
| **group*time** | 1 | 3 | 0.2923 | 0.3481 | 54 | 0.84 | 0.4049 |

| **Type 3 Tests of Fixed Effects** | | | | | | |
| --- | --- | --- | --- | --- | --- | --- |
| **Effect** | **Num DF** | **Den DF** | **Chi-Square** | **F Value** | **Pr > ChiSq** | **Pr > F** |
| **age** | 1 | 54 | 20.47 | 20.47 | <.0001 | <.0001 |
| **male** | 1 | 54 | 60.51 | 60.51 | <.0001 | <.0001 |
| **BMI** | 1 | 54 | 16.75 | 16.75 | <.0001 | 0.0001 |
| **Activity** | 1 | 54 | 0.76 | 0.76 | 0.3837 | 0.3875 |
| **group** | 1 | 54 | 0.00 | 0.00 | 0.9532 | 0.9534 |
| **time** | 2 | 54 | 10.92 | 5.46 | 0.0042 | 0.0069 |
| **group*time** | 2 | 54 | 0.80 | 0.40 | 0.6710 | 0.6729 |
